# 4-(E)-{(p-tolylimino)-methylbenzene-1,2-diol} (TIMBD) suppresses HIV1-gp120 mediated production of IL6 and IL8 but not CCL5

**DOI:** 10.1038/s41598-017-08332-z

**Published:** 2017-08-15

**Authors:** Fatma Abdalla, Anantha Nookala, Subhash B. Padhye, Anil Kumar, Hari K. Bhat

**Affiliations:** 1 Division of Pharmacology and Toxicology, School of Pharmacy, University of Missouri-Kansas City, Kansas City, Missouri 64108 USA; 2 Interdisciplinary Science and Technology Research Academy, Abeda Inamdar Senior College, Department of Chemistry, University of Pune, Pune, India

## Abstract

Human immunodeficiency virus (HIV) has been associated with inflammatory effects that may potentially result in neurodegenerative changes and a number of newer chemotherapeutic agents are being tested to ameliorate these effects. In this study, we investigated the anti-neuroinflammatory activity of a novel resveratrol analog 4-(E)-{(p-tolylimino)-methylbenzene-1,2-diol} (TIMBD) against HIV1-gp120 induced neuroinflammation in SVG astrocytes. SVG astrocytic cells were pretreated with TIMBD or resveratrol (RES) and then transfected with a plasmid encoding HIV1-gp120. The mRNA and protein expression levels of proinflammatory cytokines IL6, IL8 and CCL5 were determined. Protein expression levels of NF-κB, AP1, p-STAT3, p-AKT, p-IKKs and p-p38 MAPK were also determined. TIMBD inhibited gp120-induced RNA and protein expression levels of IL6 and IL8, but not that of CCL5 in SVG astrocytes. Moreover, TIMBD attenuated gp120-induced phosphorylation of cJUN, cFOS, STAT3, p38-MAPK, AKT and IKKs, and the nuclear translocation of NF-κB p-65 subunit whereas RES mostly affected NF-κB protein expression levels. Our results suggest that TIMBD exerts anti-inflammatory effects better than that of RES in SVG astrocytes *in vitro*. These effects seem to be regulated by AP1, STAT-3 and NF-κB signaling pathways. TIMBD may thus have a potential of being a novel agent for treating HIV1-gp120-mediated neuroinflammatory diseases.

## Introduction

Many of the human immunodeficiency virus (HIV)-associated diseases for example neurodegeneration have been potential health concerns globally^1, 2^. HIV is known to infiltrate the brain with a potential to infect different cells of the brain. In the years following HIV infection, patients show signs of neurocognitive problems termed HIV-associated neurocognitive disorders (HAND)^3–5^. Following infection of the central nervous system (CNS), viral proteins like gp120, tat and vpr are released from the infected brain cells, causing injury to the brain, and potentially leading to cognitive and motor dysfunction in the infected individuals^6, 7^. Patients suffer from memory loss, personality changes, diminished mental capacity and inability to use acquired knowledge^8–10^. Complete understanding of HIV-associated neurotoxicity has been difficult. HIV viral proteins released from infected microglia and monocytes have been generally considered as direct and indirect contributors to HAND^11, 12^. Additionally, astrocytes can also be infected with HIV leading to activation of astrocytes and subsequent release of inflammatory mediators which in turn lead to neuronal death directly or indirectly^13, 14^.

One of the major HIV viral proteins is glycoprotein120 (HIV1-gp120) that has been extensively studied for its neurotoxic contribution to the CNS^15^. Furthermore, HIV1-gp120 has been shown to induce a variety of inflammatory mediators like: TNFα, IL6, IL8 and CCL5^16–18^. Appropriate regulation of pro-inflammatory molecules is critical to inhibit or prevent inflammation. Cellular signal transduction pathways have been shown to be perturbed by HIV1-gp120^16–18^. Although chemokine receptors and their ligands play an important role in both HIV infection and immune response, how to modify these signaling processes by therapeutic intervention is still debatable and under active investigation. Reduction of cytokine production is considered an efficient therapeutic target. Cytokine expression levels are known to be controlled by their regulating transcription factors AP1, pSTAT3 and NFĸB^19, 20^. Suppression of the translocation of the above transcription factors or that of the upstream regulatory molecules can be significantly effective ways in decreasing the expression levels of cytokines.

Resveratrol (RES) is a polyphenolic phytoalexin natural compound found in many natural fruits like berries and nuts. Resveratrol has been studied extensively in different types of pathological disorders and found to be effective in study models. For example, RES has been studied for its role against oxidative stress, inflammation, cancers and many other pathological conditions^21–24^. It has been shown to produce its antioxidant effect through upregulation of Nrf2 transcription factor and consequently through the transcriptional activation of antioxidant enzymes^25^. Resveratrol has also been studied for its anti-inflammatory role and recently its effect has been suggested to be regulated by the modulation of NFĸB transcription factor and its upstream molecule Sirt1^26–28^. The lower potency of RES has however limited its use in clinical settings. Moreover, pharmacokinetic studies in healthy human subjects has revealed that RES is extensively metabolized in the liver with an oral bioavailability of <1% which further limited its success in clinical trials^29^. To improve the biological efficacy and potency of RES, we have synthesized a number of RES-analogs. One of the synthesized RES-analog 4-(E)-{(p-tolylimino)-methylbenzene-1,2-diol} (TIMBD) has been found to be effective in decreasing the proliferation of breast cancer cells^30–32^.

In the present study, we tested the effect of TIMBD against HIV1-gp120 associated neuroinflammation. Our study suggests that TIMBD has higher potency than RES in inhibiting gp120-induced inflammatory cytokine expressions in SVG astrocytes. Our study further indicates that STAT3, AP1 and NFĸB are involved in the inhibition of gp120-induced cytokine expressions by TIMBD. These results indicate that TIMBD may be a promising therapeutic candidate for decreasing HIV-associated neuroinflammation and other neurological diseases that are mediated by HIV.

## Results

We have previously reported the synthesis of 4-(E)-{(p-tolylimino)-methylbenzene-1,2-diol} (TIMBD)^31^. In the present study we investigated the effect of TIMBD against HIV-1 gp120-induced inflammation using SVG astrocytes and compared its potency/efficacy with that of RES.

### TIMBD inhibits HIV-1 gp120-induced IL6 mRNA and protein expression levels in SVG astrocytes

**Figure 1:**
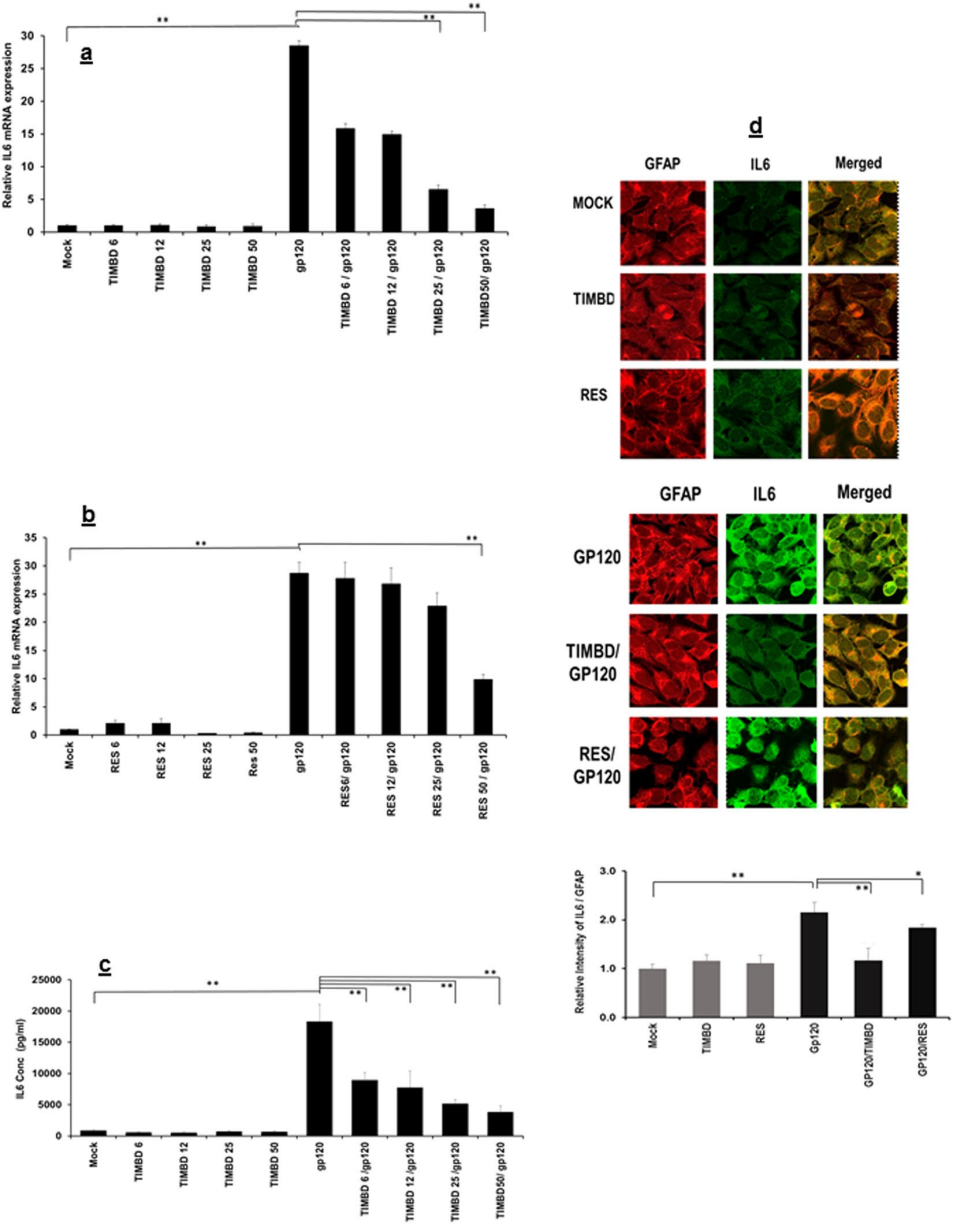
TIMBD and not RES suppresses IL6 mRNA and protein expression levels in a dose dependent manner. SVG astrocytes were treated with 6 to 50 µM TIMBD (**a**) or RES (**b**) 1 h prior to transfection and then transiently transfected with plasmid encoding HIV-1 gp120 for 5 h. The cells were harvested at 6 h and the expression levels of IL6 mRNA were determined by RT-PCR (**a** and **b**). (**c**) IL6 protein concentrations in the supernatant were measured at 48 h post-transfection by multiplex cytokine assay. (**d**) Expression levels of IL-6 and GFAP proteins were assayed by immunocytochemical methods and analyzed using confocal microscopy. The methods for Fig. 1a to d are described in the materials and methods section. The intensity of IL6/GFAP (**d**) was calculated using imageJ software. Each bar represents mean ± SE for at least 3 independent experiments, with each experiment done in triplicate. One-way Anova was used for statistical analysis and statistical significance is denoted as *(p-value ≤ 0.05) and **(p-value ≤ 0.01).

It has been previously reported that gp120 induces the mRNA and protein expression levels of proinflammatory cytokines; IL6, IL8 and CCL5 in SVG astrocytes^16–18^. It has previously been shown that gp120 induces maximal mRNA expression at 6 h and protein at 48 h post transfection^18^. In order to determine the effects of TIMBD on the expression levels of IL6 mRNA and protein, we treated the SVG astrocytic cells with doses 6 to 50 µM of either TIMBD or RES one hour prior to transfection with gp120 plasmid using serum-free medium. Then the cells were transiently transfected with 2 µg plasmid encoding HIV1-gp120 for 5 h, and the medium was changed and incubated further for 6 h and 48 h. RNA and protein was isolated using established protocols^16–18^. HIV1-gp120-induced IL6 mRNA expression levels of 28.5 ± 4.1 while treatment with TIMBD decreased expression levels of IL6 mRNA significantly in a dose dependent manner (Fig. 1a) while RES inhibited gp120-induced IL6 mRNA expression levels at 50 µM dose (Fig. 1b). Resveratrol at 50 µM dose was somewhat cytotoxic to SVG astrocytes (data not shown). We further investigated the effect of TIMBD on gp120-induced protein expression levels. We measured levels of secreted IL6 protein in supernatant 48 h following transfection of SVG cells with HIV1-gp120. We found a significant increase in IL6 protein expression levels in gp120 transfected cells 18336.9 pg/ml ± 2774.9, while TIMBD decreases IL6 protein expression levels in a dose dependent manner (Fig. 1c). Immunocytochemical (ICC) analysis was performed to measure the effect of TIMBD or RES on IL6 protein expression levels. As shown in (Fig. 1d), gp120 induced the IL6 protein expression while TIMBD, but not RES, decreased IL6 protein expression levels in SVG astrocytic cells. The intensity of IL6 relative to GFAP with HIV1-gp120 transfection was found to be 2.2 ± 0.1 fold (Fig. 1d). TIMBD treatment decreased gp120-induced IL6 intensity to 1.12 ± 0.2 fold, while RES showed less effect on IL6 intensity using confocal imaging (1.8 fold ± 0.06 fold). Thus, our results indicate that TIMBD and not RES inhibits proinflammatory cytokine IL6 at both mRNA and protein expression levels.

### TIMBD inhibits gp120-induced IL8 mRNA and protein expression levels in SVG astrocytes

**Figure 2:**
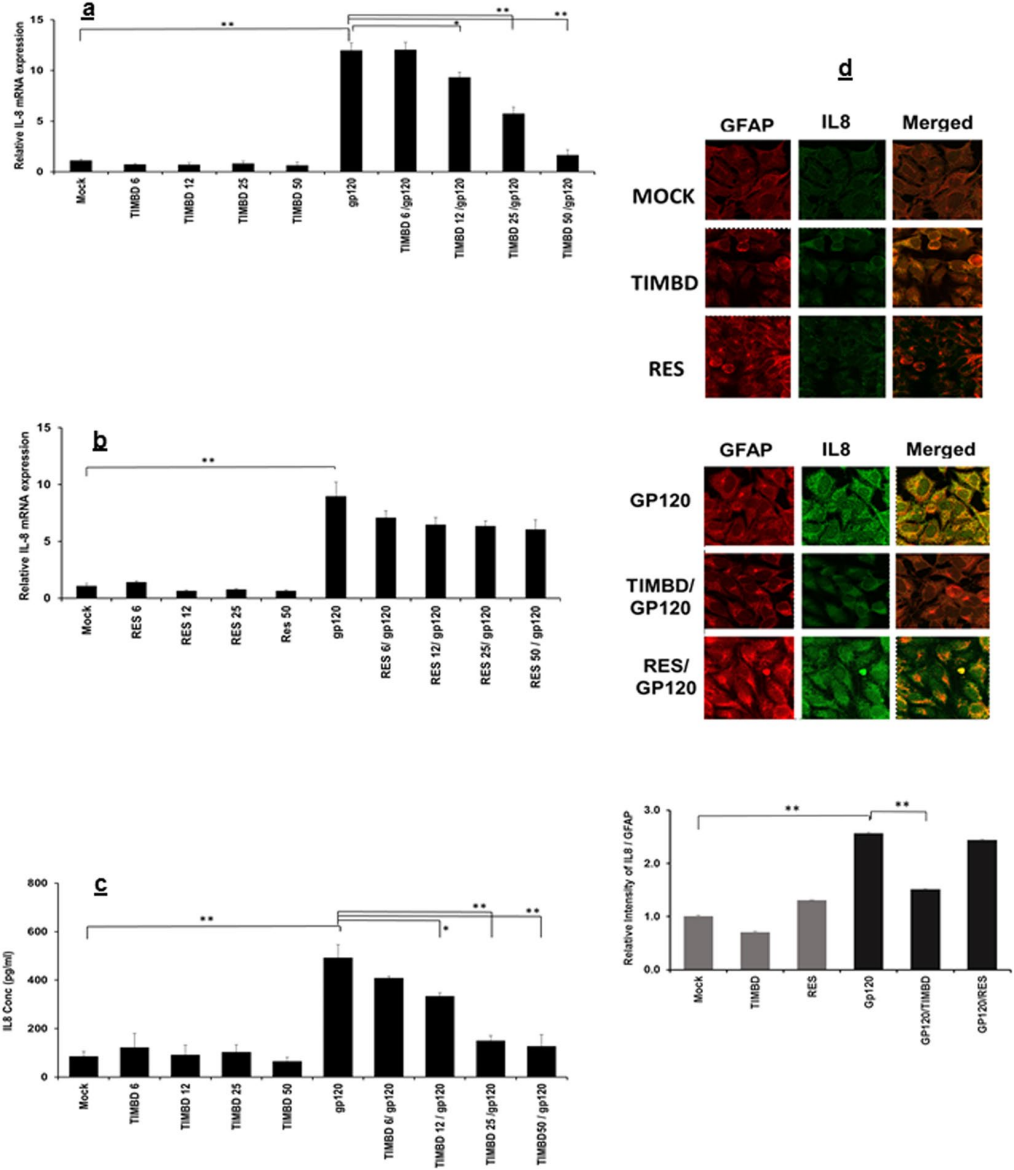
TIMBD and not RES suppresses IL8 mRNA and protein expression levels in a dose dependent manner. SVG astrocytes were treated with 6 to 50 µM TIMBD (**a**) or RES (**b**) 1 h prior to transfection and then transiently transfected with plasmid encoding HIV-1 gp120 for 5 h. The cells were harvested at 6 h and the expression levels of the IL8 mRNA were determined by RT-PCR (**a** and **b**). (**c**) IL8 protein concentrations in the supernatant were measured at 48 h post-transfection by multiplex cytokine assay. (**d**) Expression levels of IL-8 and GFAP proteins were assayed by immunocytochemical methods and analyzed using confocal microscopy. The intensity of IL8/GFAP (**d**) was calculated using imageJ software. Each bar represents mean ± SE for at least 3 independent experiments, with each experiment done in triplicate. One-way Anova was used for statistical analysis and statistical significance is denoted as *(p-value ≤ 0.05) and **(p-value ≤ 0.01).

We further identified the effect of TIMBD on IL8 mRNA and protein expression levels. It has previously been shown that gp120 induces maximal mRNA expression at 6 h and protein at 48 h post transfection^16^. In order to determine the effects of TIMBD on the expression levels of IL8 mRNA and protein, we treated the SVG astrocytic cells with doses 6 to 50 µM of either TIMBD or RES one hour prior to transfection with gp120 plasmid using serum-free medium. Then the cells were transiently transfected with 2 µg plasmid encoding HIV1-gp120 for 5 h and the medium was changed and incubated further for 6 h and 48 h. RNA and protein were isolated using established protocols as mentioned above. HIV1-gp120-induced IL8 mRNA expression levels to 11.9 ± 0.8 while treatment with TIMBD decreased expression levels of IL8 mRNA significantly in a dose dependent manner (Fig. 2a), while RES did not show any effect on gp120-induced IL8 mRNA expression levels with any dose (Fig. 2b). We further investigated the effect of TIMBD on gp120-induced protein expression levels. We measured expression levels of secreted IL8 protein in supernatant 48 h following transfection of SVG cells with HIV1-gp120. We found a significant increase in IL8 protein expression levels in gp120 transfected cells 491.7 pg/ml ± 54.3, while TIMBD decreased IL8 protein expression levels in a dose dependent manner (Fig. 2c). Immunocytochemical (ICC) analysis was performed to measure the effect of TIMBD or RES on IL8 protein expression levels. As shown in (Fig. 2d), gp120 induced the IL8 protein expression while TIMBD, but not RES, decreased IL8 protein expression levels in SVG astrocytic cells. The intensity of IL8 relative to GFAP with HIV1-gp120 transfection was found to be 2.6 ± 0.02 fold (Fig. 2d). TIMBD treatment decreased gp120-induced IL8 intensity to 1.5 ± 0.02 fold while RES showed no effect on IL8 intensity using confocal imaging 2.4 fold ± 0.01 fold. Thus, our results indicate that TIMBD and not RES inhibits proinflammatory cytokine IL8 at both mRNA and protein expression levels.

### TIMBD does not affect CCL5 in SVG astrocytes

**Figure 3:**
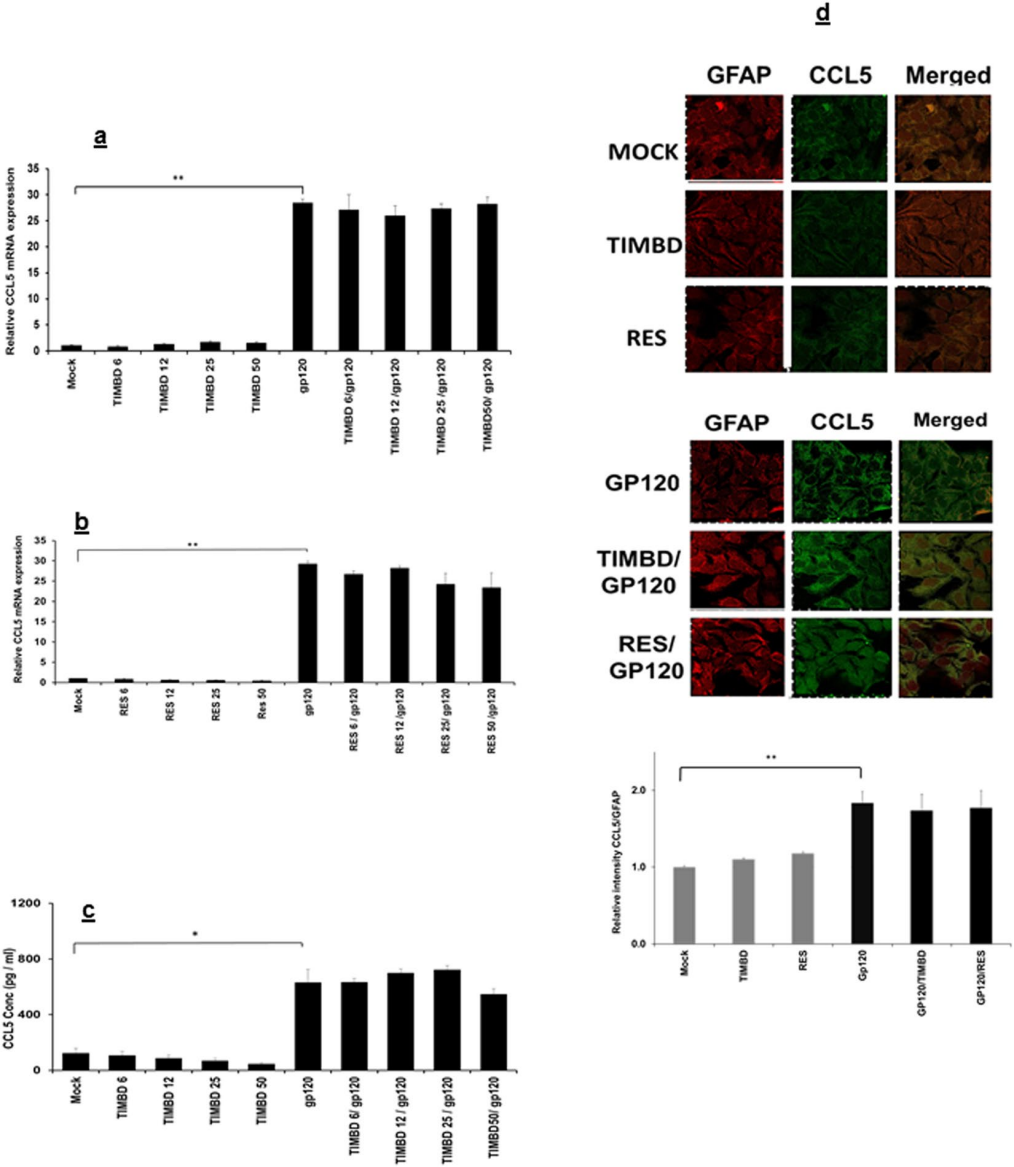
Neither TIMBD nor RES affect CCL5 mRNA or protein expression levels. SVG astrocytes were treated with 6 to 50 µM TIMBD (**a**) or RES (**b**) 1 h prior to transfection and then transiently transfected with plasmid encoding HIV-1 gp120 for 5 h. The cells were harvested at 6 h and the expression levels of CCL5 mRNA levels were determined by RT-PCR (**a** and **b**). (**c**) CCL5 protein concentrations in the supernatant were measured at 48 h post-transfection by multiplex cytokine assay. (**d**) Expression levels of CCL5 and GFAP proteins were assayed by immunocytochemical methods and analyzed using confocal microscopy. The intensity of CCL5/GFAP (**d**) was calculated using ImageJ software. Each bar represents mean ± SE for at least 3 independent experiments, with each experiment done in triplicate. One-way Anova was used for statistical analysis and statistical significance is denoted as *(p-value ≤ 0.05) and **(p-value ≤ 0.01).

We also investigated the effect of TIMBD on gp120-induced CCL5 mRNA and protein expression levels. Our studies indicate that neither TIMBD (Fig. 3a and c) nor RES (Fig. 3b) show any inhibitory effect at any dose on gp120-induced CCL5 expression at either mRNA or protein expression levels. Confocal imaging also showed the same trend with no effect of TIMBD and RES on CCL5 protein expression levels (Fig. 3d).

### TIMBD inhibits phosphorylation of STAT3, AP1 and p38 MAPK in SVG astrocytes

**Figure 4:**
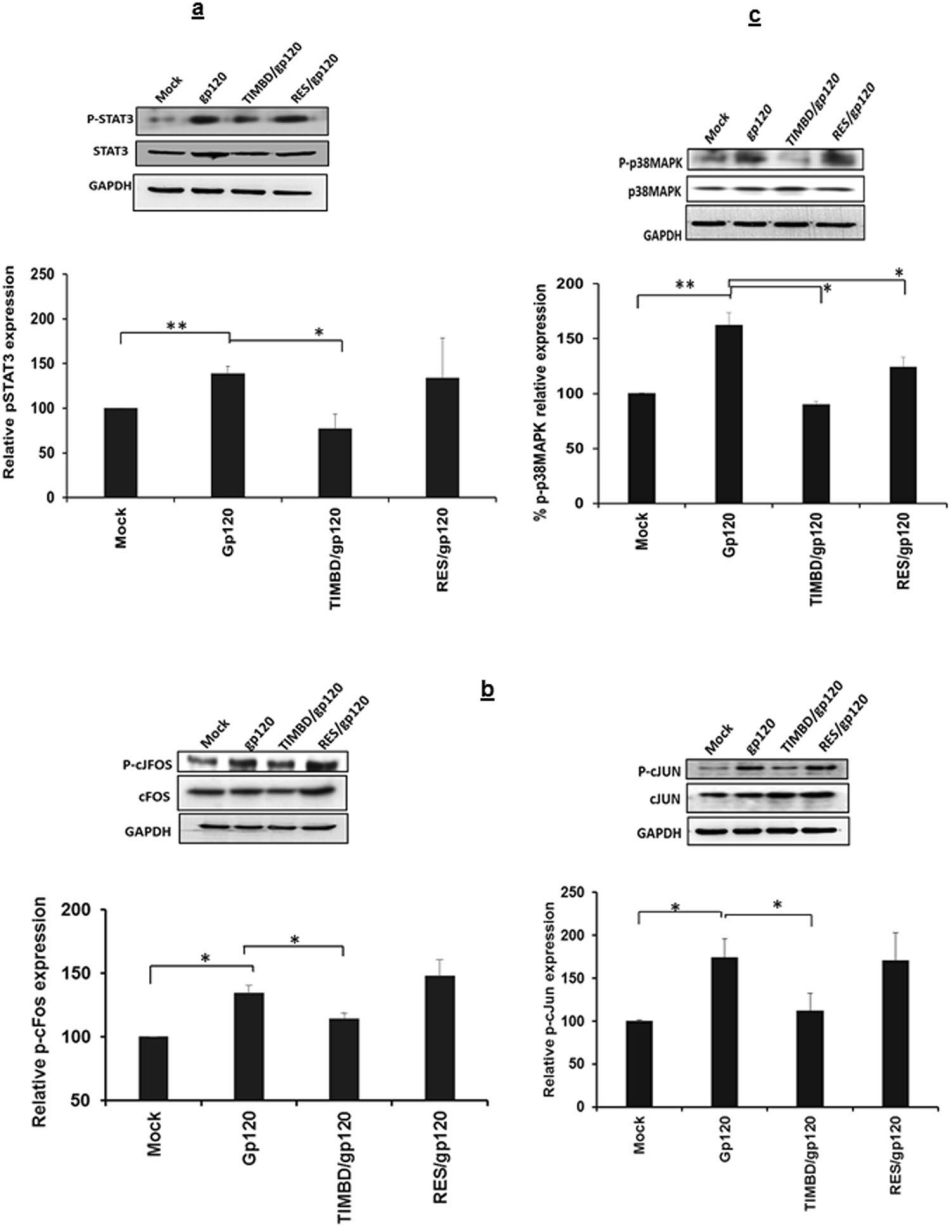
TIMBD inhibits protein expression levels of phosphorylated AP1 and STAT3 in SVG astrocytes. SVG astrocytes were treated with 50 µM TIMBD 1 h prior to transfection, then transiently transfected with plasmid encoding HIV-1 gp120 for 6 h. Protein expression levels of phosphorylated STAT3 (**a**), phosphorylated c-Jun and phosphorylated c-Fos were measured (**b**). The protein expression levels of phosphorylated p-38MAPK were determined following treatment of SVG astrocytes with 50 µM TIMBD 1 h prior to transfection with plasmid encoding HIV-1 gp120 for 1 h (**c**). The blots represented in the figures were obtained by cutting the membranes at molecular weight markers covering proteins of interest before probing them for primary and secondary antibodies. Each bar represents mean ± SE for 3 independent experiments, with each experiment done in triplicate and the western blots are representative images. One-way anova was used for statistical analysis and statistical significance is denoted as *(p-value ≤ 0.05) and **(p-value ≤ 0.01).

Phosphorylation of STAT3 and AP1 dimers (cJUN and cFOS) is known to be induced by HIV1-gp120 and that leads consequently to increase in IL6 and IL8 mRNA and protein expression levels^33, 34^. Therefore, we examined the effect of TIMBD and RES on HIV1-gp120-induced phosphorylation of STAT3 and AP1 transcription factors using western blotting, 6 h post-transfection with gp120 plasmid. We found that pre-treatment with 50 µM TIMBD decreased the HIV1-gp120-induced phosphorylation which was comparable to mock, while RES did not show any inhibitory effect (Fig. 4a). We also determined whether TIMBD affects the phosphorylation of cJUN or cFOS protein units that comprise AP1 dimer. We found that TIMBD decreased both p-cJUN and p-cFOS protein expression levels significantly while RES did not affect their expression (Fig. 4b). Additionally, we investigated the effect of TIMBD on the upstream signaling molecules regulating proinflammatory cytokines. Our results show that 1 h post-transfection of SVG astrocytes with HIV1-gp120 increased the phosphorylation of p38-MAPK (162 ± 11.3% relative to GAPDH). Treatments with TIMBD or RES did not show any effect on mock transfected cells (data not shown). TIMBD showed stronger inhibitory effect compared to RES on p38MAPK protein expression levels (Fig. 4c). Additionally, we analyzed total protein expression levels for AP1 (cJUN and cFOS) and did not find any changes in their expression levels in any of the treatments compared to mock. Although, there is a slight increase in total protein expression levels of STAT3 in gp120-trasnfected SVG astrocytes, but when we analyzed the results as phosphorylated STAT3/total STAT3 there is still an increase of phosphorylated STAT3 in gp120 transfected cells which is decreased by TIMBD but not by RES (p ≤ 0.01) compared to controls (please see Supplementary Fig. 1). These results suggest that TIMBD is capable of affecting the signaling proteins better than that of RES and thus lead to decrease in IL6 and IL8 production.

### TIMBD decreases HIV1-gp120-induced NFĸB activity in SVG astrocytes

**Figure 5:**
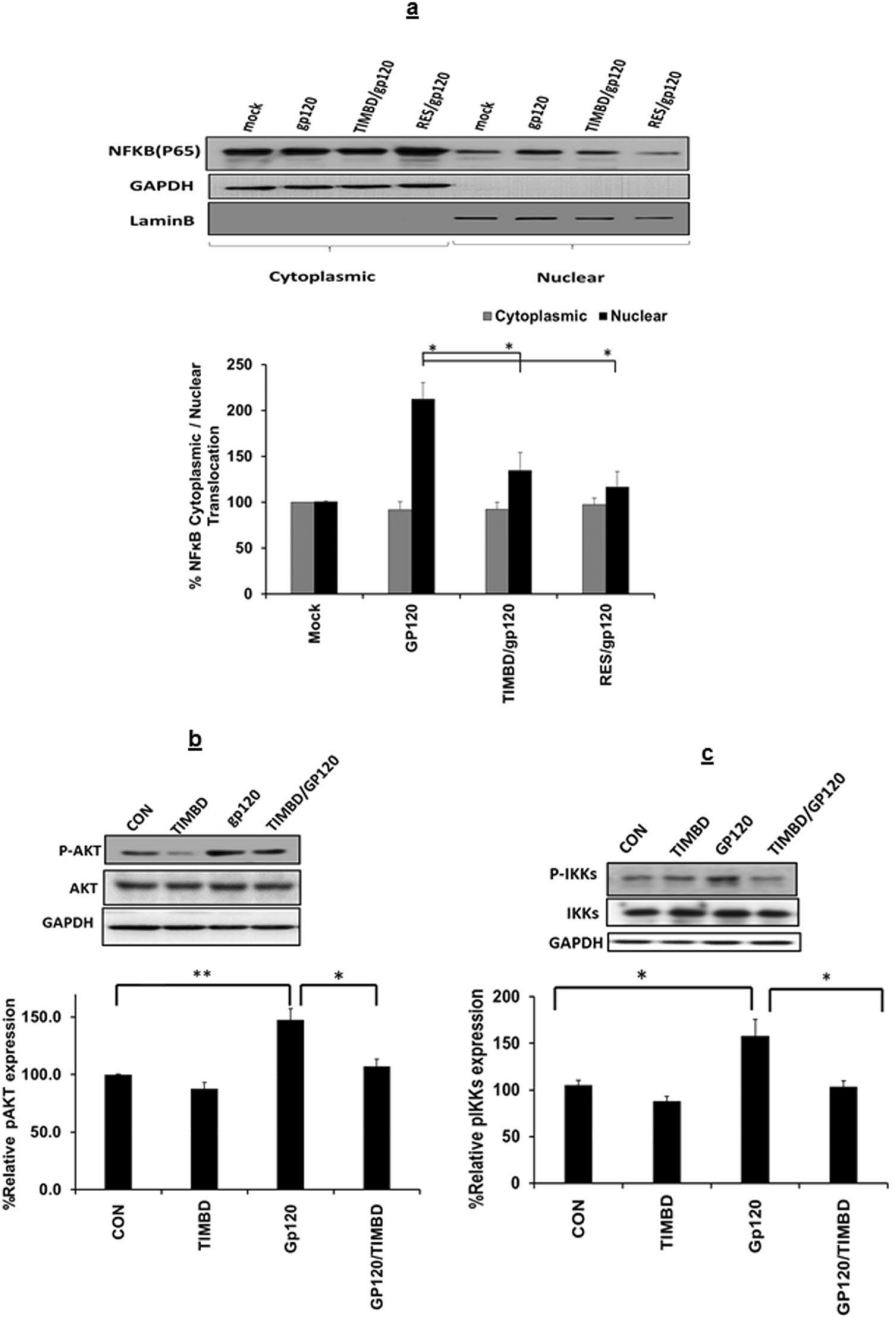
TIMBD suppresses IKKs and AKT phosphorylation and NFĸB-p65 translocation in SVG astrocytes. SVG astrocytes were treated with 50 µM TIMBD 1 h prior to transfection with plasmid encoding HIV-1 gp120 for 6 h. The presence of NFĸB-p65 subunit in the cytoplasm and the nucleus was determined (**a**). SVG astrocytes were treated with 50 µM TIMBD 1 h prior to treatment with 50 ng/ml recombinant gp120 proteins for 10 minutes. The protein expression levels of phosphorylated IKKs (**b**) and AKT (**c**) were determined using western blotting. GAPDH was used as loading control for cytoplasmic while Lamin-B was used as loading control for nuclear extracts and were used to normalize protein expression levels. The blots represented in the figures were obtained by cutting the membranes at molecular weight markers covering protein of interest before probing them with primary and secondary antibodies. Each bar represents mean ± SE for 3 independent experiments, with each experiment done in triplicate. One-way Anova was used for statistical analysis and statistical significance is denoted as *(p-value ≤ 0.05) and **(p-value ≤ 0.01).

Transcription factor NFĸB is considered a major player in the regulation of the transcription of many proinflammatory cytokines^35–38^. Previous studies have shown that RES works through NFĸB pathway to suppress inflammation in many pathological conditions^39–42^. In our study we investigated the effect of both TIMBD and RES on NFĸB translocation. Our studies indicate that gp120 increases the nuclear translocation of NFĸB p65 (210.4 ± 17.8% density) and both RES and TIMBD suppresses the NFĸB p65 subunit translocation to the nucleus (Fig. 5a). We studied the effect of TIMBD on AKT and IKKs phosphorylation which are involved in the upstream regulation of NFĸB activity. Interestingly we found that treatment of SVG ﻿astrocytes with recombinant gp120 proteins for 10 min following TIMBD pretreatment lead to significant suppression of phosphorylation of both AKT and IKKs. Additionally, we analyzed total protein expression levels for AKT and IKKs and did not found any change in their expression levels in all treatments compared to controls. Taken together the results indicated that RES might be working mostly through NFĸB, whereas TIMBD exerts its effect on multiple pathways inhibiting IL6 and IL8 mRNA and protein expression levels in SVGA cells.

## Discussion

Astrocytes are considered the most abundant non-neuronal cells in the brain which play a supportive role for the survival and functioning of the neurons and maintenance of neuronal integrity^43, 44^. Activation of astrocytes by HIV leads to oxidative stress and neuroinflammation and thus may play an important role in HIV-associated neuropathogenesis^45^. Previous studies have indicated that HIV gp120 protein associated inflammation plays a major role in neurodegeneration occurring in HIV patients. Thus efforts are being made to develop investigative molecules to inhibit HIV gp120-associated inflammation. Although neuronal death is considered the major cause for the neurodegeneration occurring in HIV patients, astrocytes are considered vital because they are involved directly and indirectly in the neuronal death. HIV gp120 has been shown to induce oxidative stress and lead to the induction of proinflammatory cytokines in astrocytes^16, 25, 46, 47^.

Resveratrol is a naturally occurring polyphenol that is studied extensively for its potential to prevent many diseases^21–24^. Resveratrol has been shown to play an important role against oxidative stress, inflammation, neurodegeneration and cancers^21–24^. Studies also have shown that RES is able to reduce inflammation and oxidative stress associated with HIV gp120 in brain cells ^25^. However RES has failed to exert its chemotherapeutic effects in many clinical trials (reviewed in^29^). This lack of efficacy has been attributed to many factors including stability or rapid metabolism of RES. To improve the efficacy of RES, we synthesized RES analog named 4-(E)-{(p-tolylimino)-methylbenzene-1,2-diol} (TIMBD). In the present study, we have shown that TIMBD has a higher potency towards inhibiting production of gp120-induced inflammatory cytokines. Since inflammation plays a vital role in neuropathogenesis, availability of TIMBD to prevent neurodegenerative diseases that affect individuals infected with HIV would be potentially very advantageous from treatment perspective. Our studies show that TIMBD is more effective than RES in inhibiting gp120-induced IL6/8 production. The potential difference may be because of the structural modifications introduced in TIMBD^31^. TIMBD has CH3 substituent at C-4 position on the B ring instead of hydroxyl group in RES which makes it more lipid-soluble and an aza-linkage (C=N) bond which gives it higher bond dissociation energy (615 kj/mole) compared to C=C bond (265 kj/mole) of Res^31^. Further studies need to be conducted to determine the structure-activity relationship.

Our results indicate that TIMBD is able to decrease gp120-mediated increase in mRNA and protein expression levels of IL6 and IL8. On the contrary, we found that RES can results in a decrease in IL6 mRNA expression levels at 50 µM. This dose was found to be cytotoxic to SVG astrocytes (data not shown). To dissect the mechanism by which TIMBD might potentially affect IL6 and IL8 expression, we determined the protein expression levels of phosphorylated STAT3 and phosphorylated dimer of AP1 (cJUN and cFOS). We observed that TIMBD, but not RES, affected the protein expression levels of phosphorylated STAT3 and phosphorylated dimer of AP1 (cJUN and cFOS), with the expression levels of both proteins being decreased. We further studied the role of MAP kinase in TIMBD-mediated inhibition of gp120-induced inflammation. We found that TIMBD decreases the expression levels of phosphorylated p38MAP kinase significantly compared to RES. We focused only on p38MAP kinase in this study as it is reported to be the most important MAP kinase in IL6/STAT3 signaling^33^. We also determined the effect of TIMBD on the nuclear translocation of NFĸB which plays an important role in promoting the transcription of IL6 and IL8. Previous studies have shown that RES produces its anti-inflammatory effects through decreasing the NFĸB translocation from cytoplasm to nucleus and could be attributed to increasing SIRT1 expression that is known as an inhibitor for NFĸB translocation. We found that both TIMBD and RES decrease NFĸB expression levels in the nucleus. These findings indicate that TIMBD is working through multiple pathways rather than only one pathway unlike RES. That may explain why TIMBD decreases IL6 and IL8 expression levels more than that of RES which showed effect only at a higher dose. Our studies demonstrate that TIMBD inhibits IL6 and IL8 but not CCL5 mRNA and protein expression levels. We have not determined the effect of TIMBD on different isoforms of p38MAPK, AKT or IKKs which may regulate CCL5. There also could be other pathways involved in CCL5 production which TIMBD does not regulate. Further studies need to be performed to study these differences.

**Figure 6:**
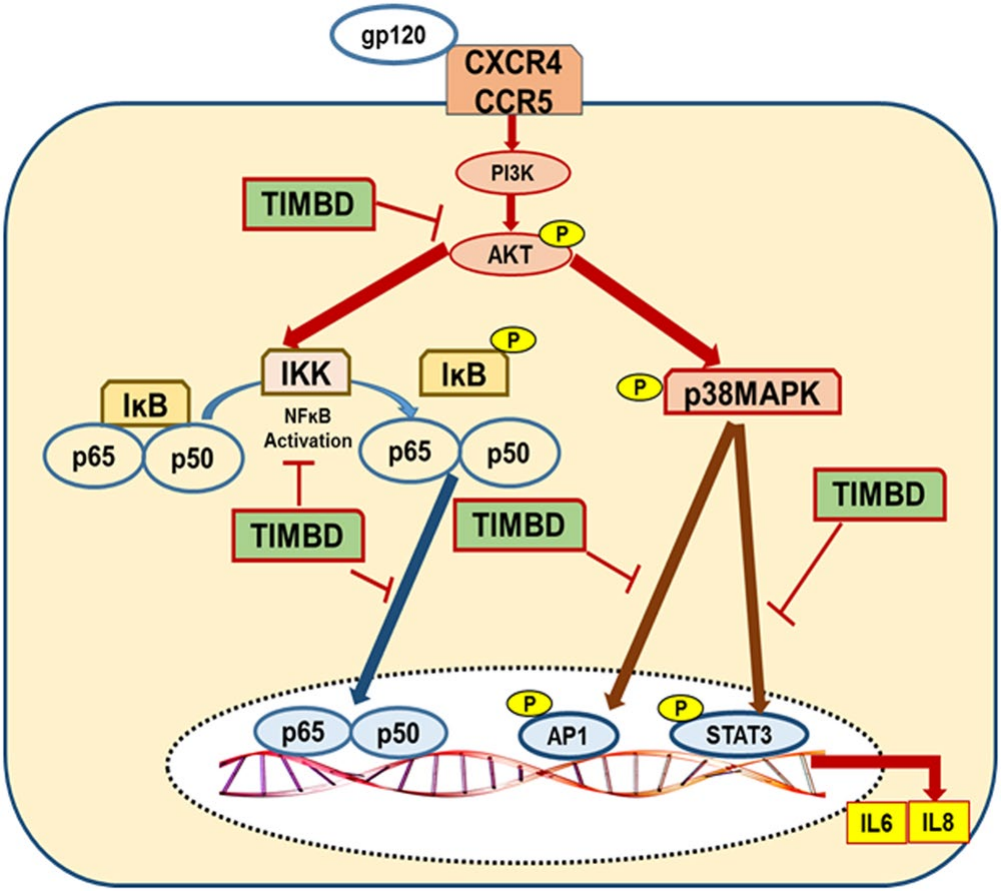
Schematic illustration of the suggested mechanism for inhibition of gp120-induced IL6 and IL8 by TIMBD in SVG astrocytes.

In summary, our results show that TIMBD decreases HIV1-gp120-associated production of IL6 and IL8 cytokines in astrocytes. Our studies further suggest that TIMBD might be working through decreasing the expression levels of phosphorylated p38MAPK and thus subsequently decreasing the phosphorylation of AP1 and STAT3 transcription factors and thus their translocation to the nucleus. TIMBD also decreases the protein expression levels of phosphorylated AKT and IKKs which are major players for NFĸB translocation from cytoplasm to nucleus leading to decrease in the expression levels of NFĸB in nucleus. These findings suggest that TIMBD might be working through multiple signaling pathways to decrease HIV-gp120 associated cytokine production in astrocytes (Fig. 6).

## Materials and Methods

### Cell Culture and chemicals

All the experiments were performed using SVG astrocytes. The cells were cultured in Dulbecco’ modified Eagle’s Medium (DMEM) that was supplemented with 10% fetal bovine serum and 1% non-essential amino acids, 1% sodium bicarbonate, 1% L-glutamine and 0.1% gentamicin. The cells in culture were maintained at 37 °C with 5% CO_2_. HIV1-gp120 (pSyngp120 JR-FL) plasmid used for transfection was originally constructed by Drs. Park and Seed (Department of Molecular Biology, and Melvin and Barbara Nessel Gene Therapy Center, Massachusetts General Hospital, Boston, MA, USA). Recombinant HIV-1 IIIB gp120 was obtained from NIH AIDS reagents program. Resveratrol was purchased from Sigma–Aldrich (St. Louis, MO). Resveratrol analog, TIMBD was synthesized and purified by us as reported previously^31^. Resveratrol and TIMBD were dissolved in dimethyl sulfoxide (DMSO) prior to treatments. The concentration of DMSO in control experiments was always 1/1000th (vol/vol) of the final medium volume. Antibodies NFĸBp-65, p-p38 MAPK, p-AKT and Lamin-B were obtained from Santa Cruz Biotechnology (Dallas, TX, USA). Antibodies for p-cJUN, p-cFOS, pSTAT3, and GAPDH and appropriate anti-immunoglobulins antibodies were obtained from cell signaling (Danvers, MA, USA).

### Transient Transfection

SVG astrocytic cells were transfected with 2 µg HIV1-gp120 plasmid transiently using Lipofectamine 2000 transfection reagent (Invitrogen, Carlsbad, CA). SVG astrocytes were plated either in 6 or 12-well plates the day prior to the experiment and allowed to adhere overnight. On the day of the experiment, cells were washed twice with phosphate-buffered saline (PBS) and then added serum-free DMEM. For TIMBD or RES treatments, cells were treated 1 hr prior to transfection and the transfection mix containing lipofectamine and optimum mixture with or without HIV1-gp120 plasmid, was added. Cells were washed with PBS twice after 5 hr of transfection, and media was replaced with serum-supplemented DMEM.

### Reverse transcription and real-time PCR

Real-time PCR was used to quantify mRNA expression levels of IL6, IL-8 and CCL5 cytokines. After treatments with RES or TIMBD, RNA from cultured SVG astrocytic cells was isolated using IBI-RNA kit reagent from Mid Sci as suggested by the manufacturer. Primer sequences and conditions have been described previously^16, 18^. The expression of hypoxanthine phosphoribosyl transferase (HPRT), a housekeeping gene, was used for quantification and standardization purposes, as reported previously^16, 18^. The expression levels of IL6, IL-8 and CCL5 relative to HPRT were determined by using the 2^−ΔΔct^ method.

### Western blotting analysis

Total protein was extracted from whole cell lysates using radioimmunoprecipitation assay (RIPA) buffer, preceded by homogenization and spinning at 10,000 rpm for 10 min. For nuclear and cytoplasmic extraction, the cells were briefly trypsinized and centrifuged at 10,000 rpm for 5 min. The cell pellet was suspended in 300 µl for cytoplasmic extraction buffer, followed by incubation on ice and washing with PBS. For nuclear extraction, 200 µl nuclear extraction buffer was added to the cell pellet, incubated on ice for 15 min and the protein collected. Protein concentration was determined using BCA kit (Pierce Biotechnology, Rockford, IL, USA). Twenty micrograms of total protein was size fractionated on a 12% SDS-polyacrylamide gel at 90 V for 3 hours, and transferred onto PVDF membranes at 100 V for 90 minutes. Membranes were blocked in 5% dry non-fat milk/PBS/0.05% Tween-20 at room temperature for 1 hr. The membranes were incubated with primary antibodies overnight at 4 °C followed by washing with PBS with 0.05% Tween-20 and incubation with secondary antibody at room temperature for 1.5 hrs. After incubation, membranes were washed and chemiluminescent detection was performed using the BM Chemiluminescence Detection kit (Roche, Indianapolis, IN) and the FluorChem HD2 Imaging system (Alpha Innotech Corporation, San Leandro, CA), with AlphaEaseFC Image Analysis software (Alpha Innotech Corporation, San Leandro, CA). Membranes probed for GPADH and Lamin-B for normalization of protein expression.

### Multiplex Protein Analysis

Cell culture supernatants collected after 48 hrs of treatment were centrifuged for 5 minutes and kept at −80 °C. Bio-Plex system (Life Science Research, Hercules, CA) and the associated Bio-Plex Manager 5.0 software was used to measure the protein expression levels of IL6, IL-8 and CCL5 in supernatants as reported previously^16, 18, 48^. Briefly, standard or supernatant samples were added to pre-prepared BioPlex plate containing BioPlex bead stock. Following that, the plate was incubated for 30 minutes at room temperature with detection antibody. After washing, phycoerythrin (PE)-conjugated streptavidin was added and incubated for 10 minutes then washed. The concentration of IL6, IL-8 and CCL5 were determined and analyzed using Bio Plex 5 software.

### Immunocytochemistry

Immunolocalization of IL6, IL8 and CCL5^48^ was performed using SVG astrocytic cells, cultured on cover slips in 6-well plates to 80% confluency. The cells were transfected as described previously for 24 hrs then Golgi stop (1 mg/ml) was added 6 hrs prior to termination of the experiment to prevent IL6, IL8 and CCL5 release from astrocytes into the cytosol. The cells were washed in PBS and fixed for 20 minutes in 1:1 (v/v) ice cold acetone and methanol followed by air drying for 5 minutes. The cells were then washed with PBS with 0.1% Triton-X100 and nonspecific binding was blocked by the incubation with 1% BSA in PBS with 0.1% Triton-X100 for 30 minutes. Cells were then incubated with mixture of antibodies (mouse anti-GFAP 1:1000, rabbit IL6 or rabbit IL8 1:500 or rabbit CCL5 1:500) overnight. The cells were then washed with PBS with 0.1% Triton-X100 twice and incubated with secondary antibodies containing Alexflour 488 labeled anti-rabbit IgG (1:1000) and Alexflour 555 labeled anti-mouse IgG (1:1000) in a dark room for 1 hr. After incubation with secondary antibody, cells were washed with PBS with 0.1% Triton-X100 three times followed by mounting the coverslips on a glass slide containing DAPI (Vector Laboratories, Burligame, CA, USA). The images were taken using confocal microscope, Leica TCS SP5 II (Leica Microsystems, Wetzler, Germany). GFAP was used as a housekeeping gene for analysis of the data.

### Statistical Analysis

The analyzed data is represented as a mean ± S.E. The analyzed results are from experiments performed in triplicates or quadruplicates. One-way ANOVA is used to analyze the data. p-value of ≤0.05 is represented with * while p-value with ≤0.01 is represented with ** and considered significant.

## Electronic supplementary material

Related Manuscript File
