# Supplementary material for: 4-(E)-{(p-tolylimino)-methylbenzene-1,2-diol} (TIMBD) suppresses HIV1-gp120 mediated production of IL6 and IL8 but not CCL5
Source: Sci Rep. 2017 Aug 15;7:8129. doi: 10.1038/s41598-017-08332-z (PMC5557832; doi:10.1038/s41598-017-08332-z)
Supplement: Supplementary file 1 — Related Manuscript File [file 41598_2017_8332_MOESM1_ESM.pdf]

4-(E)-{(p-tolylimino)-methylbenzene-1,2-diol} (TIMBD) suppresses HIV1-gp120 mediated production of IL6 and IL8 but not CCL5

Fatma Abdalla[1], Anantha Nookala[1], Subhash B. Padhye[2], Anil Kumar[1] and Hari K. Bhat[1]

[1] Division **of Pharmacology and Toxicology, School of Pharmacy, University of Missouri-Kansas City, Kansas City, Missouri 64108, USA**

[2] Interdisciplinary Science and Technology Research Academy, Abeda Inamdar Senior College, Department of Chemistry, University of Pune, India

Address for correspondence:

Hari K. Bhat, Ph.D.

Division of Pharmacology and Toxicology, School of Pharmacy

University of Missouri-Kansas City

5251 HSB, 2464 Charlotte Street, Kansas City, MO 64108

Telephone: (816) 235 - 5903

Fax: (816) 235 - 1776

Email: bhath@umkc.edu

Fatma Abdalla: fa7k3@mail.umkc.edu

Aanatha Nookala: anfh3@mail.umkc.edu

Subhash Padhye: Subhashpadhye@hotmail.com

Anil Kumar: kumaran@umkc.edu

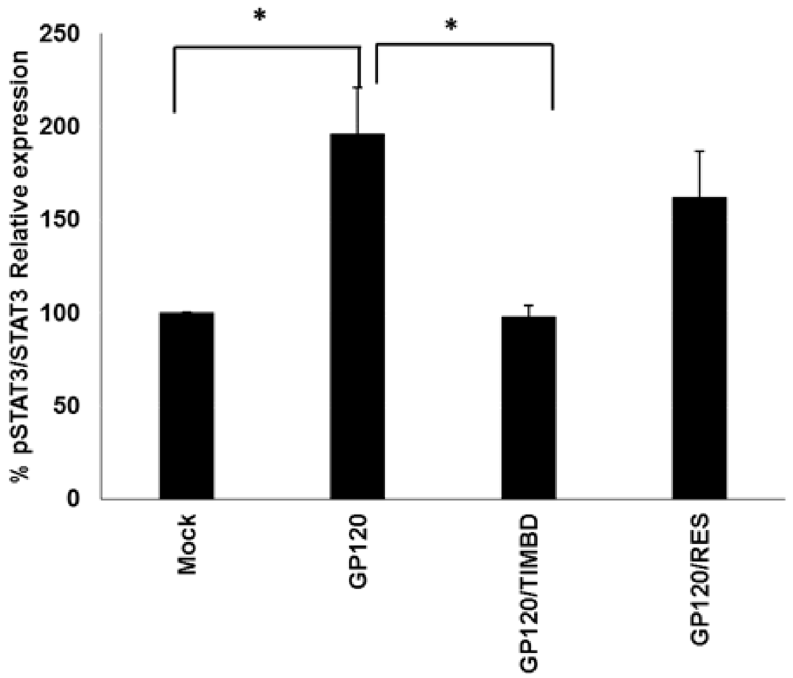

Supplementary Figure 1. TIMBD suppresses phosphorylated STAT3/total STAT3 protein expression levels in SVG astrocytes. The protein expression levels of phosphorylated STAT3 and total STAT3 were determined using western blotting. Each bar represents mean ± SE for 3 independent experiments, with each experiment done in triplicate. One-way Anova was used for statistical analysis and statistical significance is denoted as * (p-value ≤ 0.05) and ** (p-value ≤ 0.01).
